# Introduction: Non-communicable disease prevention policies in six African countries

**DOI:** 10.1186/s12889-018-5824-8

**Published:** 2018-08-15

**Authors:** Pamel A. Juma, Jennifer Wisdom

**Affiliations:** 1 African Population and Health Research Centre, Nairobi, Kenya; 2 Wisdom Consulting, New York, NY USA

Non-communicable disease (NCD) are increasing in low and mid income countries (LMICs). The greatest burden of NCDs is from cardiovascular diseases, diabetes, cancers and chronic respiratory illnesses*.* These four diseases/disease groups share a set of four risk factors: tobacco use, unhealthy diets, harmful alcohol consumption and physical inactivity. Developing NCD prevention policies that target the major risk factors is critical in LMICs that have weak health care systems and policies as well as inadequate resource capacity to implement existing health interventions. Following global commitments to address the increasing burden of NCDs, African countries have been developing policies and programs to address NCDs.

The collection of articles in this special issue titled “Non-communicable disease prevention policies in six African countries” discusses NCD prevention policies around the four major NCD risk factors. In this introductory paper, we argue that the slow process in developing effective multi-sectoral policies and programs to address NCDs in sub-saharan African countries will lead to failure to achieve global health goals such as sustainable development goals related to health. The Analysis of NCD Policies in Africa project, which was coordinated by the Africa Population and Health Research Center, is the first multi-country study to identify how sub-Saharan African countries have responded to WHO NCD prevention actions. Along with our funders, International Development Research Centre (IDRC), the primary interest in conducting this study was to examine the status of NCD prevention policies and the extent to which multi-sectoral approaches were applied in the policy development and implementation in six sub-Saharan African countries (Kenya, South Africa, Cameroon, Nigeria Malawi, and Togo). In particular, the study focused on policies addressing the WHO “best buy” interventions for NCD prevention. These interventions focus on the four major NCD risk factors, namely tobacco use, harmful alcohol consumption, unhealthy diet and physical inactivity

The articles in this issue provide a variety of perspectives as well as common themes on making positive change in NCD prevention through effective multi-sectoral policies and programs. They provide evidence on the extent of implementation of multi-sectoral action (MSA), how well countries involved multiple stakeholders across various sectors including governmental sectors, non-profits, industry, and advocacy groups in the policy processes.

A key finding in this work is that both global and local country contexts have influenced NCD policy formulation and implementation processes in the studied African countries. This is described in the first paper that focuses on the process of NCD prevention policies in Cameroon, Kenya, Malawi, Nigeria and South Africa. The factors include the historical events and country efforts as well as implementation of the WHO “best buy” interventions for NCD prevention [1]. The paper describes the development of tobacco control, alcohol control, unhealthy diet and physical activity policies in the five countries. Policy implementation challenges in these countries are also described

Another key finding is that MSA has been applied to some extent in formulating NCD prevention policies in the countries. This is described in the second paper that looked at the application of MSA in policy processes across these countries [2]. The paper describes the key governing structures that facilitate MSA as well as actions taken to ensure MSA in the countries. Recommendations to enhance MSA in African countries are also provided.

The third paper provides insights on how the WHO Framework Convention on Tobacco Control provided an unprecedented opportunity for global action against the public health effects of tobacco including non-communicable diseases [3]. This paper has broadened our understanding of how the six countries responded to the treaty to mobilize resources to develop and implement tobacco control policies. The countries utilised international regulations and commitments to accelerate policy impact on the prevention of non-communicable diseases.

The first three papers provide a synthesis of evidence across the six countries. The remaining papers address in more details, the NCD prevention policies from individual countries. Four papers address tobacco control policy development processes in Cameroon [4], Kenya [5], Nigeria [6] as well as Togo and South Africa [7]. These papers highlight the tobacco policy processes, application of multi-sectoral action in the policy development and implementation process as well as the key barriers and facilitators in developing and implementing the tobacco policies in the countries

Another paper describes Malawi’s unique approach to alcohol policy formulation and implementation [8]. Although the most recent comprehensive policy was only approved at the time data collection was almost complete, Malawi’s alcohol policy process is the most elaborately documented. Finally, the supplement article describes South Africa’s unique experience of how eliminating apartheid was associated with social justice movement to improve how the country addresses NCDs [9]. This led to multiple policies that are being implanted to address NCDs today

Together, these papers provide a comprehensive picture of how six sub-Saharan African countries are addressing policy formulation and implementation to address NCDs. They also address the ongoing need for accurate measurement to track multiple aspects of this process, including measurement of policy implementation, policy effectiveness, the extent and effectiveness of MSA. These papers provide a strong set of recommendations for sub-Saharan Africa to improve the health of its citizens.

## Abbreviations

ANPPA: Analysis of Non-Communicable Disease Prevention Policies in Africa
MSA: Multi-sectoral approach
NCD: Non-communicable diseases
WHO: World Health Organization
